# Management Impacts on Forest Floor and Soil Organic Carbon in Northern Temperate Forests of the US

**DOI:** 10.1186/1750-0680-6-17

**Published:** 2011-12-29

**Authors:** Coeli M Hoover

**Affiliations:** 1 USDA Forest Service, Northern Research Station, 271 Mast Road, Durham, NH 03824, USA

**Keywords:** forest carbon, northern hardwoods, forest management, partial harvest, clearcutting

## Abstract

**Background:**

The role of forests in the global carbon cycle has been the subject of a great deal of research recently, but the impact of management practices on forest soil dynamics at the stand level has received less attention. This study used six forest management experimental sites in five northern states of the US to investigate the effects of silvicultural treatments (light thinning, heavy thinning, and clearcutting) on forest floor and soil carbon pools.

**Results:**

No overall trend was found between forest floor carbon stocks in stands subjected to partial or complete harvest treatments. A few sites had larger stocks in control plots, although estimates were often highly variable. Forest floor carbon pools did show a trend of increasing values from southern to northern sites. Surface soil (0-5 cm) organic carbon content and concentration were similar between treated and untreated plots. Overall soil carbon (0-20 cm) pool size was not significantly different from control values in sites treated with partial or complete harvests. No geographic trends were evident for any of the soil properties examined.

**Conclusions:**

Results indicate that it is unlikely that mineral soil carbon stocks are adversely affected by typical management practices as applied in northern hardwood forests in the US; however, the findings suggest that the forest floor carbon pool may be susceptible to loss.

## Background

The development of international, state, and regional climate agreements that call for reporting and reducing the emission of greenhouse gases has led to increased interest in forest carbon inventories and a greater demand for information relating to forest carbon sequestration. In 2009, US forests are estimated to have offset 13% of national greenhouse gas emissions [1], which makes understanding the effects of forest management on forest carbon cycles a high priority. Soil carbon in particular is poorly understood; little is known about the rate of carbon accumulation, the maximum amount of carbon that can be stored in soils, effects of vegetation type, or the impact of forest management practices on soil carbon cycles. An international agreement designating the "maintenance of forest contribution to global carbon cycles" as Criterion Five in the Montreal Process for the Conservation and Sustainable Management of Temperate and Boreal Forests [2] has added to the need to understand the effects of commonly employed silvicultural techniques on key forest carbon pools.

Covington's [3] influential chronosequence study of forest floor mass in New England indicated a loss of over 50% of the forest floor mass in the 15 years after clearcutting, followed by a gradual recovery over the next 50 years. Federer [4] tested Covington's results, including an additional 14 stands, and also found that more recently cut stands had lower forest floor mass. Federer highlighted several important factors to consider, including mixing of forest floor material into the soil during harvesting and the difficulty of distinguishing the boundary of the forest floor from the mineral soil. Huntington and Ryan [5] echoed these points in another New England northern hardwoods study. Yanai et al. [6] resampled the stands used in Federer's study, and found that their data did not fit the declines predicted by the equation presented in Covington's work. Additionally, they explored some of the difficulties inherent in the substitution of space for time, and observed that differences in forest floor mass attributed to time since logging could also be explained by changes in logging techniques over the time span encompassed by Covington's chronosequence. In a study involving thinning and prescribed fire treatments applied in a network of forests across the US, Boerner et al. [7] reported no significant effects on forest floor or soil carbon stocks as a result of thinning. Some investigators have reported significant inverse relationships between thinning intensity and forest floor carbon stocks [8,9], although these studies were conducted in Norway spruce plantations with repeated thinning treatments. In the study by Jonard et al. [8], stands were treated for thirty years on a three year cutting cycle; thinning intervals in northern hardwoods are usually 15 years or longer.

Multiple review papers summarize a large number of studies on the effects of various forest management practices on forest floor and soil carbon stocks [10-13]. The results are somewhat mixed; in a few cases there appears to be a loss of carbon following harvest but much of the evidence indicates that effects are either not detectable or are short-lived, especially in the soil. One exception is when intensive site preparation resulted in extensive disturbance of the mineral soil [14], which is rare in the northern US. A meta-analysis by Nave et al. [15] included data from over 400 studies conducted around the world. Forest floor, surface soil (0-5 cm), and deep soil carbon stocks were examined, as were carbon concentrations for each respective pool. Nave et al. [15] found that forest floor carbon stocks were significantly lower (30%) in harvested sites, with different loss rates for hardwood (-36%) and softwood/mixedwood (-20%) stands. Overall, forest floor carbon concentrations declined by about 10% in harvested sites. However, no significant differences were found in carbon stocks or concentrations between harvested and unharvested stands in either shallow or deep soils, although there were some differences when soil orders were considered individually.

**Figure 1:**
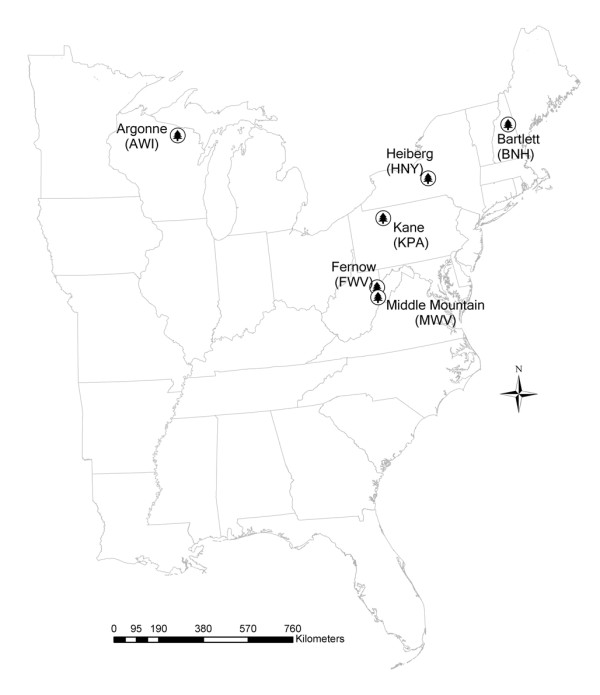
**Map showing approximate locations of each study forest**. Abbreviations following the forest names are as follows: first letter designates the forest, second two letters indicate state.

We used a common set of sampling protocols at six experimental forest sites in five northern states (see Figure 1 for approximate locations) to investigate the effects of silvicultural treatments on forest floor and soil carbon pools. The principal objective of this study was to assess the effects of partial and complete harvesting treatments on forest floor and mineral soil carbon stocks (treatments studied are listed in Table 1). Because soil carbon estimates are fairly rare in the literature, a secondary objective was to produce a set of forest floor and mineral soil carbon stock estimates for mature northern hardwood forests that can provide context for future work.

**Table 1:** Site characteristics of study forests.

Name (Abbreviation)	County/State	Forest Type Group	Major Soil Series/ Great Groups	Study Installed	Treatments Applied
Fernow (FWV)	Tucker/WV	Beech/Birch/Maple	Calvin, Dekalb/ Dystrudepts	1996	**CC**, CC with Ca and N fertilization, **No harvest**
Middle Mountain (MWV)	Randolph & Pocahontas/WV	Beech/Birch/Maple	Calvin, Dekalb/ Dystrudepts	1981	**CC w/0.93 m^2 ^residual poles**, **45% RD, 70% RD**, **No thinning**
Kane (KPA)	Elk/PA	Beech/Birch/Maple	Hazleton, Cookport, Cavode/ Dystrudepts, Fragiudults, Endoaquults	1975	**40% RD**, **70% RD, No thinning** (2^nd ^treatment in 1990)
Heiberg (HNY)	Cortland/NY	Beech/Birch/Maple	Mardin, Chippewa, Volusia/ Fragiudepts, Fragiaquepts	1970	**CC, 6.9 m^2 ^/ha (30 ft^2^/ac) **13.7 m^2^/ha (60 ft^2^/ac), **20.6 m^2^/ha (90 ft^2^/ac)**
Argonne (AWI)	Forest/WI	Beech/Birch/Maple	Wabeno, Padus, Pence/ Fragiorthods, Haplorthods	1952	**CC, 13.7 m^2^/ha (60 ft^2^/ac), 20.6 m^2^/ha (90 ft^2^/ac), No thinning** (thinning repeated in 1962, 72, 82)
Bartlett (BNH)	Carroll/NH	Beech/Birch/Maple	Marlow, Peru, Berkshire/ Haplorthods	1959	**Heavy crop tree, Light crop tree, No thinning**

CC = clearcut; m^2^/ha = residual basal area after thinning; RD = relative density, a measure of stocking. The study on the Fernow Experimental Forest is not a thinning experiment, but part of the Long Term Site Productivity Study (Adams et al. 2004) and includes harvesting and fertilization treatments; no partial harvests were applied. Forest Type Groups are as determined by US Forest Service Forest Inventory and Analysis classifications. Unless otherwise noted, treatments were applied at the time of study establishment only. Treatments in bold type are those compared in this study.

## Results

### Forest Floor

**Figure 2:**
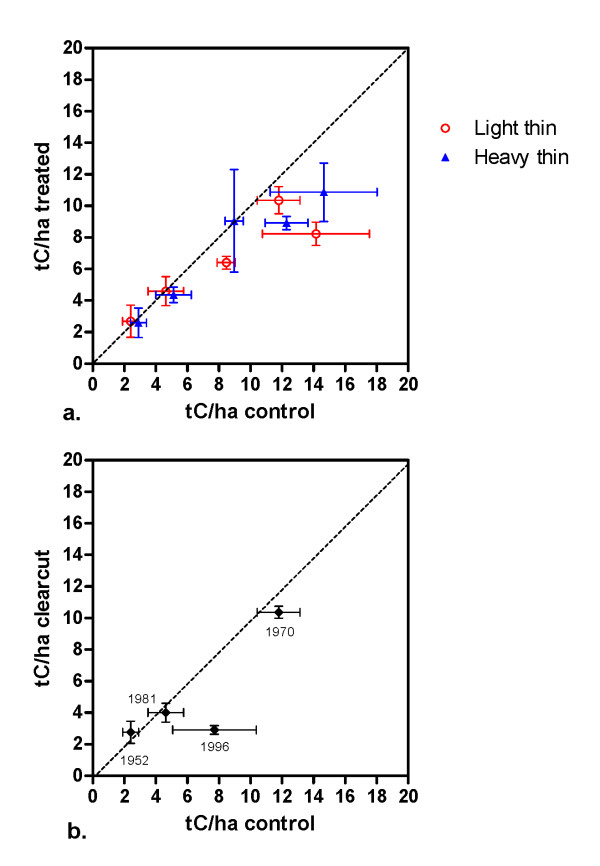
**Mean forest floor carbon stocks (tC/ha) at experimental sites**. Error bars indicate standard error of the mean (X error = control values, Y error = treated values). (a) Lightly thinned, heavily thinned, and unthinned control plots. (b) Clearcut and uncut control plots; year of cut is listed by each point.

Average forest floor carbon stocks of control plots were variable across the study region, although values for thinned and unthinned plots fell in the same range for each site (Table 2). Comparisons between stands that were lightly thinned, heavily thinned, or unthinned revealed no overall statistically significant differences (p = 0.46) between treatments. For the sites where a clearcut harvest was applied, forest floor carbon stocks were not significantly different from uncut plots (p = 0.47) across the study. Only one clearcut site, the most recent, (6 years prior to sampling) showed a large difference between forest floor carbon stocks in clearcut and control plots (Table 2, Figure 2); clearcut plots averaged 4.8 tC/ha less the uncut controls. The overall mean of forest floor carbon concentration across all untreated plots was 40.7%, with a 95% confidence interval of 38.2-43.2%. Carbon concentrations did not differ significantly among thinned and unthinned plots (p = 0.936) with values averaging from 39.3-39.9% across all treatments. However, there was a 9% difference in forest floor carbon concentrations between clearcut and control plots, with mean values of 37.8 and 41.5%, respectively; this was not statistically significant (p = 0.101).

**Table 2:** Forest floor and soil carbon stocks for each forest, by treatment intensity.

Site	State	Treatment	Forest Floor C Stocks (t/ha)	Soil C Stocks 0-5 cm (t/ha)	Soil C Stocks 0-20 cm (t/ha)	Total Forest Floor + Soil C Stocks (t/ha)
Fernow	WV	Clearcut	2.9 (0.27)	25.1 (1.76)	70 (1.4)	73 (1.6)
		Control	7.7 (2.65)	18.6 (2.05)	62 (4.6)	70 (4.0)

Middle Mountain	WV	Clearcut w/0.93 m^2 ^residual in poles	4.0 (0.6)	26 (4.75)	78 (9.3)	82 (9.9)
		Heaviest thin	4.4 (0.49)	17.8 (4.27)	68 (4.5)	73 (4)
		Lightest thin	4.6 (0.91)	18.5 (1.56)	61 (3.7)	66 (4)
		Control	4.6 (1.13)	27.1 (4.57)	77 (11.3)	81 (10.6)

Kane	PA	Heaviest thin	9.1 (3.25)	16.2 (0.53)	56 (0.4)	65 (2.8)
		Lightest thin	6.4 (0.4)	16.3 (0.42)	54 (3.4)	59 (3.2)
		Control	8.5 (0.58)	23 (1.52)	65 (3.6)	74 (4.2)

Heiberg	NY	Clearcut	10.4 (0.37)	18.6 (1.07)	61 (2.7)	71 (2.4)
		Heaviest thin	8.9 (0.43)	17.3 (1.29)	55 (3.1)	64 (3.5)
		Lightest thin	10.4 (0.87)	20.4 (2.4)	63 (1.4)	73 (0.7)
		Control	11.8 (1.35)	18.3 (0.26)	63 (1.5)	75 (0.2)

Argonne	WI	Clearcut	2.8 (0.70)	9.7 (2.04)	32 (2.8)	34 (2.6)
		Heaviest thin	2.6 (0.93)	11 (2.48)	33 (2.5)	35 (1.8)
		Lightest thin	2.7 (1.02)	16.1 (4.69)	41 (10.7)	43 (10.1)
		Control	2.4 (0.51)	13.5 (4.89)	39 (10)	41 (9.5)

Bartlett	NH	Heaviest thin	10.9 (1.85)	13.6 (1.29)	53 (7.1)	64 (8.2)
		Lightest thin	8.2 (0.73)	14.4 (2.23)	55 (6.5)	64 (6.2)
		Control	14.2 (3.39)	13.2 (1.31)	55 (4.3)	69 (7.1)

Value shown is the mean of the plot values (parentheses indicate standard error of the mean). The Heiberg study did not have formal control plots; temporary plots were established in an uncut buffer zone adjacent to the study area and used as controls. Totals may not agree with sum of category values due to rounding

### Mineral Soil

**Figure 3:**
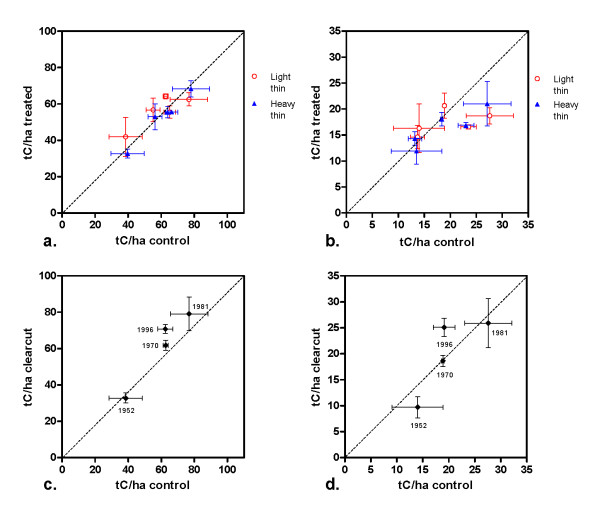
**Mean soil carbon stocks (tC/ha) at experimental sites**. Error bars indicate standard error of the mean (X error = control values, Y error = treated values). (a) Lightly thinned, heavily thinned, and unthinned control plots, 0-20 cm depth. (b) Lightly thinned, heavily thinned, and unthinned control plots, 0-5 cm depth. (c) Clearcut and uncut control plots, 0-20 cm depth; year of cut is listed by each point. (d) Clearcut and uncut control plots, 0-5 cm depth; year of cut is listed by each point. Note that scales differ.

Soil carbon stocks in the upper 20 cm of mineral soil in the control plots were variable across the study region, although most fell between 55-65 tC/ha; error estimates also varied between and within sites (Table 2, Figure 3). Overall, carbon stock estimates for the 0-20 cm depth increment were not significantly different between thinning treatments (p = 0.387), with control plots averaging 59 (± 4.4) tC/ha and mean stock values of 54.9 (± 3.1) and 52.8 (± 3.7) tC/ha for lightly and heavily thinned plots, respectively. For the surface (0-5 cm) soils, at one site (the Kane Experimental Forest, in Pennsylvania) surface soil carbon in both thinning treatments was significantly lower than in the control plots by about 6.5 tC/ha, a difference of more than 20%. (Table 2). However, on the whole, no significant differences were detected across treatments (p = 0.249).

In the clearcutting treatments, carbon stocks to 20 cm averaged 59.9 (± 5.8) tC/ha in control plots and 58.3 (± 5.7) tC/ha in the clearcut plots (Table 2, Figure 3); this was not a statistically significant difference (p = 0.842). Surface (0-5 cm) soil carbon in clearcut and control plots was similar, with mean values of 19.3 (± 2.3) and 19.5 (± 2.3) tC/ha, respectively (Table 2, Figure 3).

## Discussion

In this study, no significant differences were detected between treated and untreated plots in either the forest floor or soil carbon pools. The intrinsic variability of forest floor mass and soil chemical properties presents challenges when interpreting the results of statistical tests; differences of 20% or more can be obtained from sample points within the same plot. Combined with the low replication often unavoidable in forest management studies, a common problem in management effect experiments is a lack of statistical power to detect differences [16]. The use of *post-hoc *power analysis is often recommended as a way to assist in interpreting results, although this approach has been subject to criticism (for an example, see Hoenig and Heisey [17]).

Given the challenges inherent in detecting changes in forest floor and soil properties, summarizing the data graphically provides a useful tool for evaluating the outcomes of statistical tests. Figure 2a shows the forest floor carbon stocks in control plots and their standard errors plotted on the X axis, with the corresponding values from the thinned plots and their associated errors plotted on the Y axis. Each point represents the mean value for a particular forest; points falling below the 1:1 line indicate that stocks are higher in control plots than in thinned plots. Most points fall close to the line, with a few below, indicating that while there is no overall treatment effect, in some cases forest floor carbon stocks may be lower in plots that were thinned (although the error values for the control means are large). Figure 2b presents the same information for clearcutting, the most extreme treatment. The plot supports the finding of no overall significant differences; the point falling below the line is from the most recently harvested site, which was cut about 6 years prior to sampling.

While there is no obvious relationship of treatment intensity and forest floor carbon stocks, one trend is evident in Figure 2a. It is generally true that since decomposition is related to climatic variables, forest floor mass should increase with increasing latitude, although litter chemistry also plays a role [18,19]. In this study forest floor carbon pools are higher at the northernmost forests and lower at the more southerly sites, but a notable exception is the Argonne Experimental Forest in northern Wisconsin, where forest floor mass was lower than might be expected (this forest is represented by the leftmost point in both Figures 2a and 2b). This is most likely due to sizeable numbers of invasive earthworms. While no population estimates were made, earthworms were commonly observed during sampling on the Argonne. Rapid increases in earthworm populations and the resulting effects on the forest floor in the Lake States have been documented [20,21].

Soil carbon stocks to 20 cm are shown in Figures 3a (thinning treatments) and 3c (clearcuts) and support the finding of no differences across treatments, with points falling near the 1:1 line, and slightly above for most of the clearcut sites. Given the difficulty of detecting small changes in soil carbon stocks against a large background it is helpful to examine the surface soils, which are shown in Figures 3b (thinning treatments) and 3d (clearcuts). Here as well, most points fall near the line and no treatment effect is evident, although at the most recent clearcut site surface soil carbon averaged 6.5 tC/ha higher than in the control sites (about 12%).

**Figure 4:**
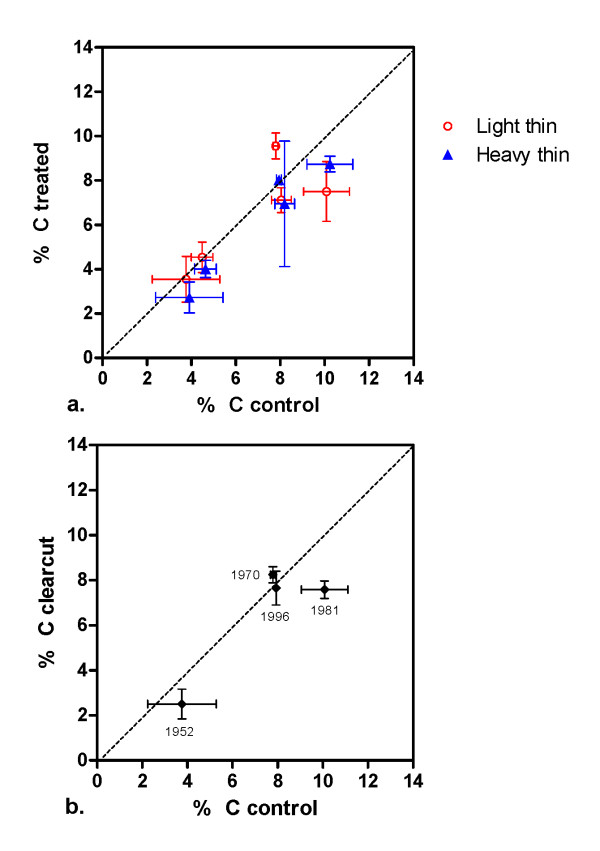
**Mean soil carbon concentration 0-5 cm (% C) at experimental sites**. Error bars indicate standard error of the mean (X error = control values, Y error = treated values). (a) Lightly thinned, heavily thinned, and unthinned control plots. (b) Clearcut and uncut control plots; year of cut is listed by each point.

Because we are interested in the size of the carbon pools the most relevant quantity is carbon content (stock). Soil carbon stock estimates are the product of bulk density and carbon concentration measurements, and a change in one property may mask a change in the other. Carbon concentrations in the surface (0-5 cm) soil are plotted in Figure 4a for thinned plots and Figure 4b for clearcut treatments and show a similar outcome to the surface carbon stocks, supporting the finding of no overall differences related to treatments.

Although the forest floor carbon stocks showed a trend of increasing values from southern to northern sites with the exception of the Argonne Experimental Forest, this same trend was not evident for any of the soil properties measured. Similar to the forest floor results, carbon stocks in both depth increments and surface soil carbon concentrations were lowest at the Argonne Experimental Forest, which is represented by the leftmost point in every figure. Earthworm activity can lower bulk density as well as redistribute organic matter and carbon throughout the soil profile [22]. While carbon concentrations are usually highest in the surface soil, this may not be the case where earthworms are prevalent. If a substantial population of non-native earthworms is encountered at a site, sampling protocols may need to be adjusted.

The results reported here, no systematic declines in soil carbon stocks or concentrations in plots subjected to partial or complete harvesting, agree with the findings of the large meta-analysis conducted by Nave et al. [15]. Other studies in the region report similar results: Morris and Boerner [23] in Ohio found no differences in carbon stocks in heavily thinned stands at one site and increased soil carbon at another, and Mattson and Smith [24] reported no differences in soil organic matter content or concentration across a chronosequence of cut and uncut plots in West Virginia. Mattson and Smith [24] did find that harvested sites had significantly lower forest floor organic matter content than control sites, which agrees with the findings of Nave et al., Covington, and Federer [15,3,4]. While we did not detect significant differences in forest floor carbon stocks between treated and control sites, in a few forests carbon stocks were higher in the control plots (Figures 2a and 2b), and there was a difference of about 9% between the mean forest floor carbon concentration in clearcut and control sites.

## Conclusion

This study found no significant differences in mineral soil carbon stocks between northern hardwood forest stands that had been previously clearcut and uncut controls, with average values (to 20 cm depth) of 58.3 (± 5.7) and 59.9 (± 5.8) tC/ha, respectively. Carbon stocks in the top 5 cm of mineral soil averaged about 19 tC/ha for both clearcut and control sites, with standard errors of 2.3 tC/ha for each. In the partial harvest treatments, there were no significant differences in soil carbon stocks between lightly thinned, heavily thinned, or unthinned plots for either depth increment, nor were there overall differences in soil carbon concentrations across treatments. On the whole, no significant differences were detected in forest floor carbon pools between clearcut and control plots. In the partial harvest treatments, a few forests had greater forest floor carbon stocks in control plots, although mean forest floor carbon storage displayed no overall trend across treatments. Given the results presented here, it is unlikely that mineral soil carbon stocks are adversely affected by typical management practices as commonly applied in northern hardwood forests in the region; however, the findings suggest that the forest floor carbon pool may be susceptible to loss.

## Methods

### Site description

Study sites included six forests in five northern states: West Virginia (WV), Pennsylvania (PA), New York (NY), Wisconsin (WI), and New Hampshire (NH). Four of the sites are US Forest Service Experimental Forests, one is a research area on the Monongahela National Forest, and one is a university research forest (SUNY-ESF). At five of the sites (Argonne - WI, Bartlett - NH, Heiberg - NY, Kane - PA, and Middle Mountain - WV), ongoing long-term thinning studies were sampled to assess the effects of silvicultural thinning treatments of different intensities on soil and forest floor carbon stocks. The sixth site, Fernow - WV, is not a thinning study but is part of the nationwide long-term soil productivity study (LTSP) network [25,26]. Table 1 provides additional detail about each site, and Figure 1 indicates the approximate location of each forest. Further details on each of the forests that are part of the Experimental Forest Network may be found in Adams et al. [27]. While the treatments at each forest vary, all of the studies are replicated, are variations on the randomized block design, and include a range of thinning intensities; four sites included plots that had been clearcut. Some forests have been treated more than once. Generally, three replicates of each treatment were part of the original study design, though in a few cases there were only two replicates of a particular treatment. Plot size varied across the studies but generally ranged from 0.16 to 0.24 ha (0.4-0.6 ac). While the dominant species varied across the sites, all are classified in the beech/birch/maple forest type group as defined by the US Forest Service Forest Inventory and Analysis Program (FIA).

### Field and laboratory methods

Sampling took place in the spring and summers of 2001-2003; all the plots on a given forest were sampled over a three or four day period. Forest floor samples included all organic material above the mineral soil, and were collected on a systematic grid across the plot using a 25 cm^2 ^sampling frame following the general method outlined by Harmon et al. [28]. Four samples were taken at each plot and were not composited; all material was oven dried at 48°C and weighed, then coarsely ground. Carbon concentrations were determined on homogenized subsamples by dry combustion. Mineral soil samples were collected on a systematic grid of twelve points across the plot (although sometimes conditions required collecting fewer cores), with a slide impact hammer corer following the methods used by the US Forest Service Forest Inventory and Analysis Program [29]. Each sample was 5 cm × 20 cm, split into 0-5, 5-10, and 10-20 cm increments. Soils were oven dried at 105°C, sieved, and analyzed by dry combustion for carbon concentration. Separate samples were taken at each site to determine bulk density for use in calculating carbon stocks. Because of the spatial variability of coarse fragments and the difficulty of obtaining accurate site-specific values for this property, no adjustments were made for coarse fragment volume and all calculations were made based on the assumption of rock-free soil volume. Although this results in stock estimates that are likely to be higher than actual values, estimates of coarse fragment volume can have a large effect on estimates and introduce a sizeable amount of error.

### Calculations and Statistics

All data were checked for outliers with the Grubbs outlier test. Soil carbon content (mass) was calculated as the product of bulk density and carbon concentrations. Forest floor carbon stocks were the product of forest floor mass per unit area and carbon concentration. All analyses were conducted with SigmaStat software (SPSS Incorporated). Where the data met the assumptions of normality and homogeneity of variances, t-tests were used for comparisons between clearcut and control sites, and partial harvest treatments were compared with ANOVA. In a few instances, data were non-normally distributed and simple transforms did not achieve normality. In these cases, a distribution-free rank-sum test was applied.

## Competing interests

The author declares that they have no competing interests.
